# Engineering a functional tendon-bone interface: mechanisms and prospects of magnesium alloys in promoting neurotized regeneration via modulating stem cell-nerve interplay

**DOI:** 10.3389/fmed.2026.1832217

**Published:** 2026-05-20

**Authors:** Zhu Xinke, Yue Hao, Sun Zhengming, Dong Xianghui, Ling Ming

**Affiliations:** 1 Department of Orthopedics, Shaanxi Provincial People’s Hospital, Xi’an, China; 2 Key Laboratory of Basic and Clinical Transformation of Bone and Joint Diseases in Shaanxi Province, Xi’an, China; 3 Graduate School of Xi’an Medical University, Xi’an, China

**Keywords:** magnesium alloy, mesenchymal stem cells, neurotized regeneration, proprioception, tendon-bone interface

## Abstract

**Objective:**

Conventional strategies for tendon-bone interface (TBI) repair primarily focus on structural healing, often overlooking the essential processes of neural regeneration and proprioceptive recovery required for functional restoration. This review aims to explore the potential of biodegradable magnesium (Mg) alloys and the released magnesium ions (Mg^2 +^) in establishing a “Mg^2 +^-Stem Cell-Nerve” axis as a novel strategic foundation for achieving neurotized regeneration at the TBI.

**Methods:**

This review provides a narrative synthesis of the existing literature on the roles of Mg^2 +^ in regulating stem cell functions and promoting neural regeneration. A multidimensional perspective integrating “immunity-metabolism-nerve” interactions was adopted to dissect the underlying synergistic molecular mechanisms. Furthermore, the design of intelligent Mg-based implants predicated on this theory was discussed.

**Results:**

Analysis of the existing evidence suggests that Mg^2 +^ may act as a pivotal bioactive signal, independently and synergistically regulating stem cell behavior and neural regeneration processes, thereby supporting the proposal of a conceptual “Mg^2 +^-Stem Cell-Nerve” axis. This proposed axis could theoretically synchronize structural repair and neural re-innervation of the TBI. Based on this mechanism, the design of intelligent Mg-based implants demonstrates significant potential for achieving spatiotemporally precise modulation.

**Conclusion:**

Biodegradable Mg alloys, through the proposed “Mg^2 +^-Stem Cell-Nerve” axis, offer a promising paradigm for advancing TBI healing from structural integration toward neurotized functional regeneration. However, clinical translation remains at an early stage, requiring further validation in large-animal models, resolution of degradation control challenges, and rigorous long-term safety and efficacy evaluation. Although the proposed “Mg^2 +^-Stem Cell-Nerve” axis provides a novel integrative framework, it is important to note that its full sequential and closed-loop operation currently remains a working hypothesis derived from synthesizing fragmented pairwise evidence from disparate model systems, rather than a fully established pathway directly validated in the TBI microenvironment.

## 1 Clinical challenges in tendon-bone interface repair and the importance of reinnervation

**FIGURE 1:**
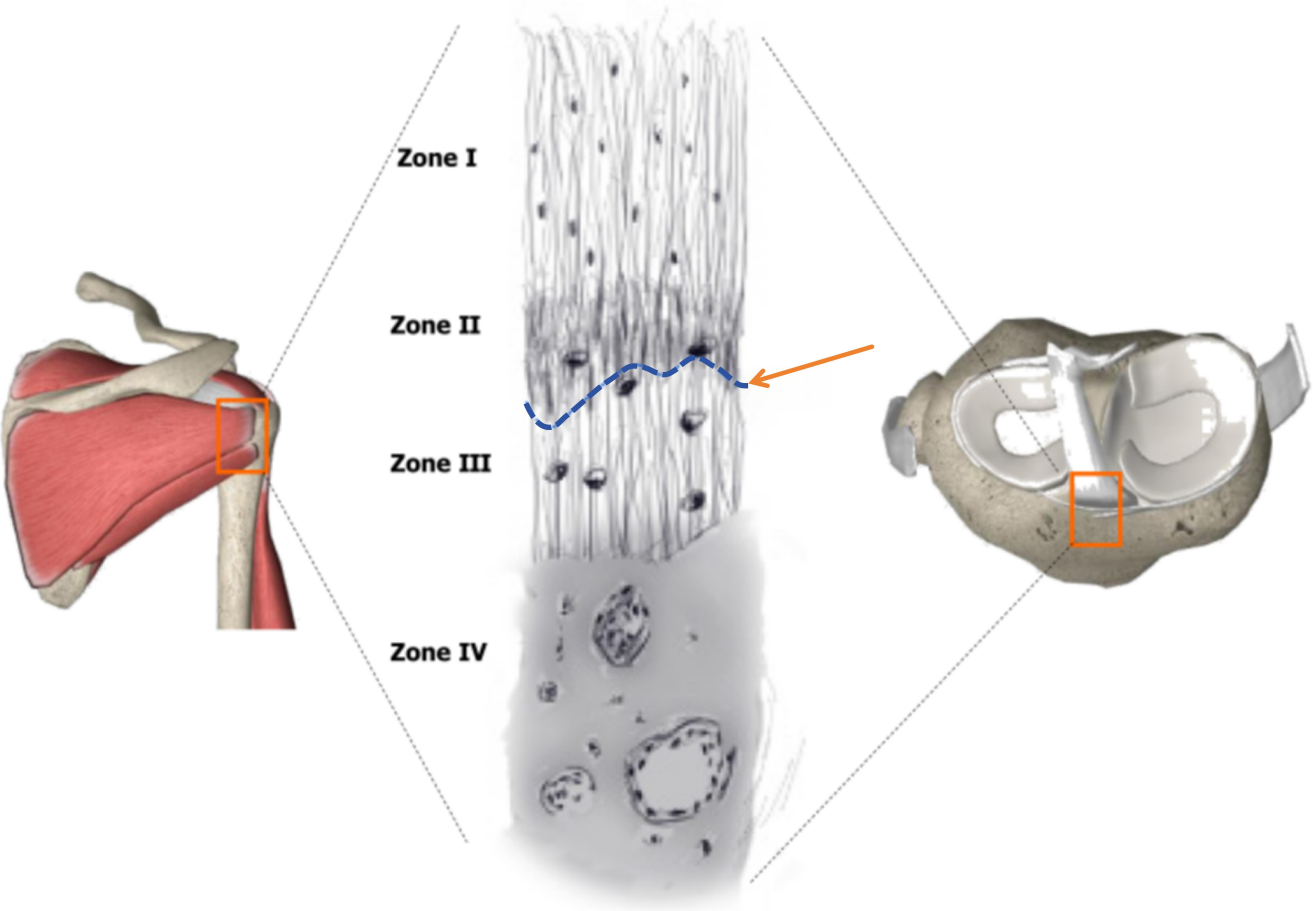
Structure of the tendon-bone insertion. Zone I consists of ligament. Zone II comprises nonmineralized fibrocartilage. Zone III is composed mineralized cartilage. Zone IV consists of bone. Tidemark between Zone II and Zone III (black arrow) is shown. Adapted from Figure 1 in Xu et al. (1), World J Stem Cells, 13(7), 753–775. Reprinted with permission from the publisher under a Creative Commons Attribution License.

The tendon-bone interface (TBI), such as the rotator cuff and anterior cruciate ligament (ACL), is a composite structure integral to both “mechanical load transfer” and “sensory modulation.” Its gradient structure, transitioning from tendon (rich in type I collagen) to fibrocartilage (rich in type II collagen and proteoglycans) to bone (mineralized tissue), provides the anatomical basis for effective load transmission and stress distribution (Figure 1). The fibrocartilage zone, a key component of this gradient, functions as a biomechanical buffer and stress transition zone, rather than a simple “Sharpey’s fiber”-like direct insertion of collagen bundles into bone (1, 2). Simultaneously, the dense distribution of nerve endings (e.g., Ruffini endings, Pacinian corpuscles) in this region precisely regulates proprioception by sensing mechanical changes, collectively maintaining joint dynamic stability and motor coordination (3, 4).

However, the natural healing process following TBI injury struggles to reconstruct this sophisticated “structure-function” unit, representing a multi-stage but incomplete repair cascade. It initiates with an inflammation phase dominated by macrophages, which clear necrotic tissue and release repair signals via pathways like TLR/NF-κB. This is followed by a proliferation/repair phase, where cells such as mesenchymal stem cells (MSC, primarily derived from bone marrow, synovium, or peritendinous tissues) are recruited under the guidance of signals like TGF-β and SDF-1. These MSC differentiate into fibroblasts, synthesizing abundant type III collagen to form granulation tissue, while Schwann cells (SCs) are activated to support axonal sprouting. The final remodeling/maturation phase involves mechanically stimulated collagen remodeling, yet neural regeneration is significantly delayed and insufficient. This leads to the core defect of neo-scar tissue: structural disorganization (lacking the natural gradient) and sparse neural innervation (5, 6).

Current mainstream repair strategies (e.g., growth factor delivery, cell therapy, traditional biomaterial implantation) predominantly focus on collagen deposition and bone ingrowth, often failing to synchronously reconstruct the gradient structure and neural innervation (7–9). This directly contributes to suboptimal clinical outcomes. Epidemiological data indicate that graft-tunnel healing failure remains a leading cause of reoperation after ACL reconstruction, with revision rates reaching up to 7.1% in specific high-risk populations (10). For rotator cuff repair, even post-surgery, re-tear rates for large or massive tears remain as high as 20%–40% (11). Typical cases of repair failure manifest not only as an increased tendon-bone gap or re-tear on imaging but, more fundamentally, as incomplete functional recovery. Clinical research and biomechanical testing reveal that failed cases often feature neo-interfaces dominated by structurally disorganized fibrous scar tissue, lacking the normal mechanical gradient. Crucially, proprioceptive function remains impaired, with over 60% of patients demonstrating increased position sense errors and diminished dynamic balance postoperatively, a factor widely considered key to elevated re-injury risk (12). Postoperative TBI healing thus faces multiple challenges: weak structural integration, loss of proprioception, and high re-injury rates (13). Therefore, how to rebuild neural innervation alongside structural repair has emerged as a central issue for enhancing functional recovery (14, 15). This underscores the core tenet of “neurotized tissue engineering”: actively modulating biological signals to achieve synergistic advancement of TBI structural regeneration and neural reinnervation (16).

Mesenchymal stem cells, often derived from bone marrow, synovium, or peritendinous tissues, possess self-renewal and multipotent differentiation capabilities, and are key “effector cells” mediating structural regeneration (17). To clarify the concepts and ensure terminological consistency throughout this review, we define the key terms as follows. “Neurotized regeneration” (synonymous with “innervated regeneration”) refers to a healing outcome that simultaneously achieves reconstruction of structural integrity and restoration of functional neural innervation; this is the preferred term in this review. “Neural regeneration” specifically refers to the regeneration and extension process of neural components themselves, such as neurons, axons, and Schwann cells. “Reinnervation” describes the functional restoration of nerve supply to a target tissue. Throughout this review, these terms are used consistently as defined above, and “innervated regeneration” appears only in this definition. Based on this cellular foundation, neurotized regeneration in the TBI context presents a clear interactive logic: regenerated nerve endings not only restore proprioception but also, via released neuropeptides (e.g., Calcitonin Gene-Related Peptide, CGRP), actively regulate MSC osteogenic differentiation and H-type vessel angiogenesis through pathways like CALCRL/PKA, forming a proactive “neuro-vascular-bone” collaborative regeneration network (4). Within this context, biodegradable magnesium alloys, leveraging their triple advantage of “mechanical support - bioactivity - degradation compatibility,” emerge as an ideal platform for modulating the “Stem Cell-Nerve” interplay and achieving neurotized regeneration of the TBI.

## 2 Unique attributes of magnesium alloys: a multifunctional bioactive platform beyond mechanical support

Biodegradable magnesium alloys (e.g., pure Mg, Mg-Zn, Mg-Ca alloys) demonstrate comprehensive advantages distinct from traditional inert materials in tendon-bone interface repair. The Mg^2 +^ released during their degradation actively modulates the regenerative microenvironment, enabling a functional shift from “passive support” to “active regulation” (Table 1). It is essential to clarify that the core bioactive agent discussed in this review is the ionic form (Mg^2 +^) released from the degradation of magnesium-based materials, not the alloying elements themselves (although elements like Zn also possess bioactivity).

**TABLE 1:** Multidimensional bioactive roles and mechanisms of magnesium ions (Mg^2 +^) in tendon-bone interface regeneration.

Target/category	Key biological effects	Proposed mechanisms/pathways	Functional outcome in TBI	Evidence source
Mesenchymal stem cells (MSC)	Promotes proliferation, migration, and osteogenic/tenogenic differentiation	Activates PI3K/Akt, Erk pathways; upregulates CXCR4; modulates Runx2, SCX	Provides “seed cells” for structural repair; establishes a foundation for regeneration	*In vitro* (38, 39); bone defect models (73)
Neurons and axons	Promotes neurite/axonal outgrowth; exerts neuroprotection	Activates PI3K/Akt; upregulates guidance molecules (e.g., Sema5b); antagonizes NMDA receptors	Directly drives neural regeneration and functional reinnervation	Sciatic nerve injury models (44, 45); *in vitro* (23, 40)
Schwann cells (SCs)	Promotes proliferation, migration, activation; enhances NGF synthesis/secretion	Modulates inflammatory microenvironment; potential pathway specifics under investigation	Supports axonal guidance, re-myelination, and creates a pro-regenerative niche	*In vitro*; sciatic nerve models (45, 52)
Immune cells (macrophages)	Promotes M2 polarization; exerts anti-inflammatory effects	Reduces pro-inflammatory cytokines (TNF-α, IL-1β, IL-6, IFN-γ, MCP-1); increases anti-inflammatory cytokines	Creates an anti-inflammatory, pro-healing microenvironment, protecting SCs and neurons	*In vitro*; general inflammation studies (62, 63)
Neuropeptide signaling	Stimulates CGRP release from sensory neurons	Activates cAMP/CREB and CALCRL/PKA pathways in MSC and endothelial cells	Forms a “neuro-vascular-bone” feedback loop, enhancing osteogenesis and vascularization	Fracture healing model (periosteum) (88, 89)
Integrated microenvironment	Creates a neurotrophic, anti-inflammatory, and pro-regenerative milieu	Combined effects on above cells; enhanced secretion of BDNF, NGF, GDNF, etc., from activated MSC	May synergistically support both structural and neural components of TBI healing.	Synthesis from multiple systems (4, 15, 85, 86)

“Evidence source” indicates the primary model systems from which supporting data are derived. Direct evidence from tendon-bone interface (TBI) models remains limited for most entries; see Section “5.1 Comprehensive review of existing evidence and directions for deepening mechanistic research” for discussion of knowledge gaps.

### 2.1 Synergistic mechanical compatibility and electroactivity

Compared to bio-inert materials like titanium alloys and polyetheretherketone (PEEK), magnesium alloys possess significant advantages in active biological regulation. Even compared to bioactive materials like calcium phosphate ceramics, magnesium alloys not only release pro-osteogenic signals but also offer mechanical properties matching bone tissue, controllable degradability, and favorable electroactive properties (18, 19). This characteristic is particularly important for TBI repair, as successful healing requires not only structural regeneration of bone and tendon but also reconstruction of neural function. Compared to another class of biodegradable metals, zinc-based alloys, magnesium has more mature *in vivo* metabolic pathways, a wider safety margin, and more substantial evidence supporting its direct promotive effects on the nervous system. A systematic review of preclinical models concluded that magnesium-based fixation enhances tendon-graft healing, further highlighting the unique value of magnesium alloys in TBI repair (20).

Regarding core properties, the density (1.74–2.0 g/cm^3^) and elastic modulus (41–45 GPa) of magnesium alloys are close to those of human bone, effectively mitigating the “stress-shielding effect” caused by the stiffness mismatch with titanium alloys (elastic modulus ∼110 GPa) and reducing the risk of bone resorption (21). More critically, their degradation rate can be precisely tuned through alloying and processing techniques to achieve synchronization with the TBI regeneration timeline: providing tensile strength of 200–300 MPa in the early repair phase to stabilize the injury environment, and subsequently degrading gradually as new tissue forms and replaces it. This perfectly aligns with the physiological logic of “repair–degradation–regeneration,” laying the foundation for tendon-bone structural regeneration.

Following nerve injury at the TBI, disruption of electrical signal conduction directly leads to functional loss. Substantial evidence confirms that enhancing the electroactivity of biomaterials is an effective strategy for promoting neural function reconstruction (22–25).

Worthy of specific comparison is that, for the goal of neural regeneration, emerging conductive polymers (e.g., PEDOT:PSS) or piezoelectric scaffolds (e.g., poly-L-lactic acid, PLLA) hold the core advantage of providing exogenous or motion-induced electrical stimulation, directly modulating the electrophysiological activity of neural cells and promoting axonal growth (26–28). However, such polymeric materials often face limitations including insufficient mechanical strength (especially initial strength), potential inflammation from acidic degradation products, and the lack of inherent pro-osteogenic/pro-tenogenic ionic signals (29–31). In contrast, magnesium itself possesses good conductivity; it can provide an electrical stimulation pathway for damaged nerves and the muscles they innervate, thereby promoting neural regeneration (32). Its incorporation into repair systems can enhance material electroactivity, offering crucial support for the repair and reconstruction of neural function at the TBI (33). Therefore, for complex requirements like the TBI that demand simultaneous fulfillment of load-bearing, structural regeneration, and neural innervation reconstruction, magnesium alloys demonstrate more comprehensive, integrated potential. Future development will likely involve convergent innovation rather than mutual replacement, such as constructing magnesium-conductive polymer composites to synergistically leverage the multiple advantages of mechanical support, ion release, and electrical stimulation, ultimately achieving dual repair of tendon-bone structure and neural function.

However, it is crucial to distinguish between the biological effects of Mg^2 +^ ions and the concurrent physicochemical changes brought about by magnesium alloy degradation. Degradation involves hydrogen evolution, local alkaline shifts, and possible release of other alloying elements, all of which may influence the regenerative microenvironment. While Mg^2 +^ is considered a key signaling molecule, many studies report the net outcome of these combined factors. Future research should aim to decouple ionic effects from degradation-related physicochemical changes to precisely attribute specific biological outcomes.

### 2.2 Biosafety: metabolizable without accumulation

Magnesium ions, as the fourth most abundant cation in the human body, is an essential electrolyte that can be normally metabolized and excreted via the kidneys, posing no risk of accumulation (34, 35). Simultaneously, as an essential cofactor for over 300 enzymes in the human body, it activates key enzyme systems such as ATPase and alkaline phosphatase, playing a central role in energy metabolism and bone mineralization (35). Furthermore, well-designed magnesium alloy degradation products do not elicit significant immune rejection, providing a biosafety guarantee for their long-term application at the TBI.

### 2.3 Multidimensional bioactivity: the signaling function of Mg^2 +^

Beyond its enzymatic cofactor role, Mg^2 +^ plays a key part in cellular signal transduction (36).

At the stem/progenitor cell level, Mg^2 +^ acts as an effective mitogen, precisely regulating the proliferation, migration, and lineage-specific differentiation of stem cells through activation of key signaling pathways like PI3K/Akt (37). Regarding migration, it upregulates the CXCR4 receptor on mesenchymal stem cells, enhancing SDF-1α/CXCR4 axis-mediated chemotactic migration, while also recruiting MSC to the injury site via chemokines like TGF-β1 and PDGF-BB (38). In differentiation, it directs MSC toward the osteogenic/tenogenic lineages required for the TBI by modulating transcription factors such as Runx2 (osteogenic) (39).

At the nervous system level, Mg^2 +^ directly promotes axonal growth and modulates neurotransmitter systems. Mg^2 +^ participates in membrane phospholipid structure, intracellular signaling, and myelination, and can regulate the transmission of important neurotransmitters like dopamine and serotonin (5-HT) (23, 40). Numerous studies further confirm that magnesium directly promotes axonal growth and stem cell proliferation (22, 39).

Building upon this multidimensional bioactivity, this review proposes the core hypothesis of the “Mg^2 +^-Stem Cell-Nerve” Interplay Axis: Mg^2 +^ serves as the central biological signal, directly orchestrating stem cell behavior to promote structural regeneration of the TBI on one hand, while supporting nerve regeneration by modulating stem cell secretory functions and acting directly on neural cells on the other, ultimately achieving synergistic functional repair of the tendon-bone interface.

## 3 Direct and indirect roles of magnesium ions in nerve regeneration

It is important to note at the outset that the majority of evidence regarding the direct neuroregenerative effects of Mg^2 +^ derives from peripheral nerve injury models (e.g., sciatic nerve crush or transection) and *in vitro* studies using neuronal or Schwann cell cultures. Direct evidence generated within the tendon-bone interface (TBI) microenvironment remains limited. Extrapolation of these findings to TBI healing should therefore be made with appropriate caution, and the application of this evidence to the TBI context is presented here as a hypothesis-generating synthesis.

### 3.1 Direct promotion of axonal growth and neuronal activity

Axonal regeneration is a core component of functional recovery in peripheral nerves (41). Biofunctional metal ions such as magnesium (Mg), zinc (Zn), and calcium (Ca) are essential for promoting neural regeneration by modulating axon and Schwann cell activity (42).

Magnesium ions enhances neurite outgrowth in a concentration-dependent manner by activating the Phosphatidylinositol 3-kinase (PI3K)/Akt signaling pathway and upregulating axonal guidance molecules like Sema5b (43). *In vivo* studies using Mg^2 +^-loaded conduits in rat sciatic nerve injury models also confirmed significant axonal regeneration and functional recovery (43). This indicates that controlled release of Mg^2 +^ is crucial for promoting axonal regeneration and nerve repair.

Another study employing degradable magnesium wires as an ion-release platform in a sciatic nerve compression model provided direct evidence of Mg^2 +^ promoting axonal regeneration: morphologically, it significantly increased the length and number of regenerating axons; molecularly, it upregulated the expression of axonal growth-related markers (e.g., Growth-Associated Protein 43 - GAP-43, Neurofilament) (44). These results confirm that Mg^2 +^ acts directly on injured neurons to promote axonal growth and elongation.

### 3.2 Promotion of Schwann cell proliferation and functional activation

Most human nerve fibers are myelinated, with myelin sheaths formed by Schwann cells (45). Schwann cells are glial cells constituting the peripheral nervous system. They not only participate in myelination but also, following peripheral nerve injury, rapidly proliferate, divide, and secrete various protein molecules to maintain a supportive environment for axonal growth, thereby facilitating neural self-repair and regeneration (44). After nerve injury, Schwann cells dedifferentiate, proliferate, and migrate to the lesion site, where they promote the repair process by forming Büngner bands – structures guiding axonal regeneration (42, 46). Moreover, they secrete abundant endogenous neurotrophic factors (NTFs), including Nerve Growth Factor (NGF) (44). NGF plays a promotive role in the repair and regeneration of injured peripheral nerves, inducing neurite outgrowth, preventing neuronal degeneration and apoptosis, and thereby facilitating neural regeneration and repair (47). NGF is reported to act directly on axonal fiber growth rather than on the neuronal soma itself (48, 49).

Research has found that Mg^2 +^ not only enhances the survival and proliferation of SCs but also significantly promotes their migration, guiding axonal regeneration, resulting in regenerated axons with higher density and greater length, collectively driving nerve fibers to extend into the injured area and achieve defect repair (42, 50). Furthermore, studies have found that appropriate concentrations of magnesium ions can promote SC proliferation and stimulate SC synthesis and secretion of NGF (51).

Li et al., using a sciatic nerve injury model, confirmed magnesium’s role in promoting myelinated axonal regeneration post-injury. After implanting magnesium wires at the injury site, the expression of nerve growth factor (NGF), p75 neurotrophin receptor, and Tyrosine receptor kinase A (TrkA) mRNA was upregulated, and the number of transected nerve fibers and regenerating axons significantly increased (44). Therefore, degrading Mg^2 +^ can promote SC synthesis and secretion of NGF, which in turn further promotes neurite outgrowth, establishing a positive feedback loop that provides a favorable environment for neural repair at the tendon-bone interface.

### 3.3 Neuroprotection and microenvironment regulation: NMDA receptor antagonism and anti-inflammatory effects

#### 3.3.1 Neuroprotective functions of magnesium ions

The neuroprotective effects of magnesium ions on the central nervous system have been extensively studied. Clinically, magnesium sulfate is used to prevent cerebral palsy in preterm infants. Research by Wolf et al. revealed that antenatal magnesium sulfate administration to women threatening preterm delivery between 24 and 32 weeks of gestation reduces the risk of moderate-to-severe cerebral palsy, demonstrating neuroprotection (52). Furthermore, magnesium has been found to improve functional neurological recovery in patients with global brain ischemia associated with cardiac surgery and cardiac arrest (53). Although this evidence comes from central nervous system studies, it suggests a potential neuroprotective mechanism that may also be relevant in the peripheral neural environment of the TBI.

Nerve injury is often accompanied by damage to nutrient vessels, leading to nerve ischemia and hypoxia (54). When neurons are deprived of normal oxygen levels, mitochondrial damage and energy metabolism disruption occur, reducing ATP production and enzyme activity (55). This activates N-methyl-D-aspartate (NMDA) receptors on the cell membrane, which are specific calcium channels (56). After hypoxic injury, opening of NMDA receptor channels causes massive intracellular calcium influx and Ca^2 +^ overload, further exacerbating energy metabolism disruption, leading to oxygen free radical production and oxidative phosphorylation, generating large amounts of excitatory amino acids glutamate and GABA, ultimately resulting in ischemic neuronal death (57).

Research indicates that Mg^2 +^ can mitigate secondary damage after neural injury by modulating cell function, antagonizing NMDA receptors, and countering calcium influx, thereby exerting neuroprotective effects (58). Under stress, magnesium ion levels drop, leading to excessive opening of calcium channels, calcium influx, and subsequent cell swelling and apoptosis. Therefore, Mg^2 +^ supplementation can block the NMDA receptor ion channel via charge interaction, inhibiting calcium entry into cells, while also counteracting injury-induced changes in membrane permeability and the neurotoxic effects of calcium (59).

#### 3.3.2 Magnesium ions suppress inflammation to promote peripheral nerve repair

The anti-inflammatory effects described here are largely attributed to Mg^2 +^ based on controlled ion supplementation studies. However, in the context of degrading magnesium alloys, local pH elevation and hydrogen release may also modulate inflammatory responses, potentially in opposing directions. Therefore, the observed *in vivo* anti-inflammatory outcomes likely represent a net effect of multiple degradation products, not solely Mg^2 +^.

Inhibiting the inflammatory response can promote neural regeneration by preventing Schwann cell (SC) apoptosis (60). Magnesium ions facilitate peripheral nerve repair by suppressing inflammation. Magnesium promotes macrophage polarization toward the M2 phenotype, increasing anti-inflammatory cytokine production while reducing pro-inflammatory cytokine levels (61). This shift helps prevent chronic inflammation, protects Schwann cells from apoptosis, and supports motor neuron survival after sciatic nerve injury. Magnesium modulates this process by shifting macrophages from the inflammatory M1 to the anti-inflammatory M2 phenotype, thereby creating a favorable environment for nerve regeneration (62).

Pan et al. found that a high-magnesium diet significantly increased plasma and nerve tissue magnesium concentrations, improving neurobehavioral and electrophysiological function, and reducing the accumulation of inflammatory cells (e.g., macrophages) and the expression of inflammatory factors. Compared to high concentrations, a low magnesium environment may produce the opposite effect, enhancing inflammation in injured nerves. High magnesium supplementation promoted the expression of anti-apoptotic proteins Bcl-2 and Bcl-XL, subsequently downregulating the expression of activated caspase-3 and cytochrome c, thereby reducing Schwann cell apoptosis (63). Magnesium supplementation significantly decreased pro-inflammatory cytokines, such as Tumor Necrosis Factor-α (TNF-α), Interleukin-1β (IL-1β), IL-6, and Interferon-γ (IFN-γ), thus promoting neural regeneration. Additionally, magnesium reduced the release of MCP-1, a key chemokine in inflammation, preventing chronic inflammation. High magnesium supplementation inhibited the expression of Monocyte Chemoattractant Protein-1 (MCP-1) and Regulated on Activation, Normal T Cell Expressed and Secreted (RANTES), thereby suppressing macrophage aggregation and myelin clearance (64).

In addition, a comprehensive review by Uyanikgil et al. further summarizes conserved neuroregenerative and neuroprotective signaling pathways underlying functional recovery after peripheral nerve injury, providing important background support for the neural repair mechanisms discussed in this review (65).

## 4 Core chapter: constructing the “Mg-Stem Cell-Nerve” interactive axis

The following sections synthesize evidence from multiple research systems–including peripheral nerve injury models, bone fracture healing studies, and *in vitro* investigations–to construct a conceptual framework for neurotized TBI regeneration. Where evidence is drawn from non-TBI models, this is explicitly noted, and the inferential nature of its application to the TBI context is acknowledged.

**FIGURE 2:**
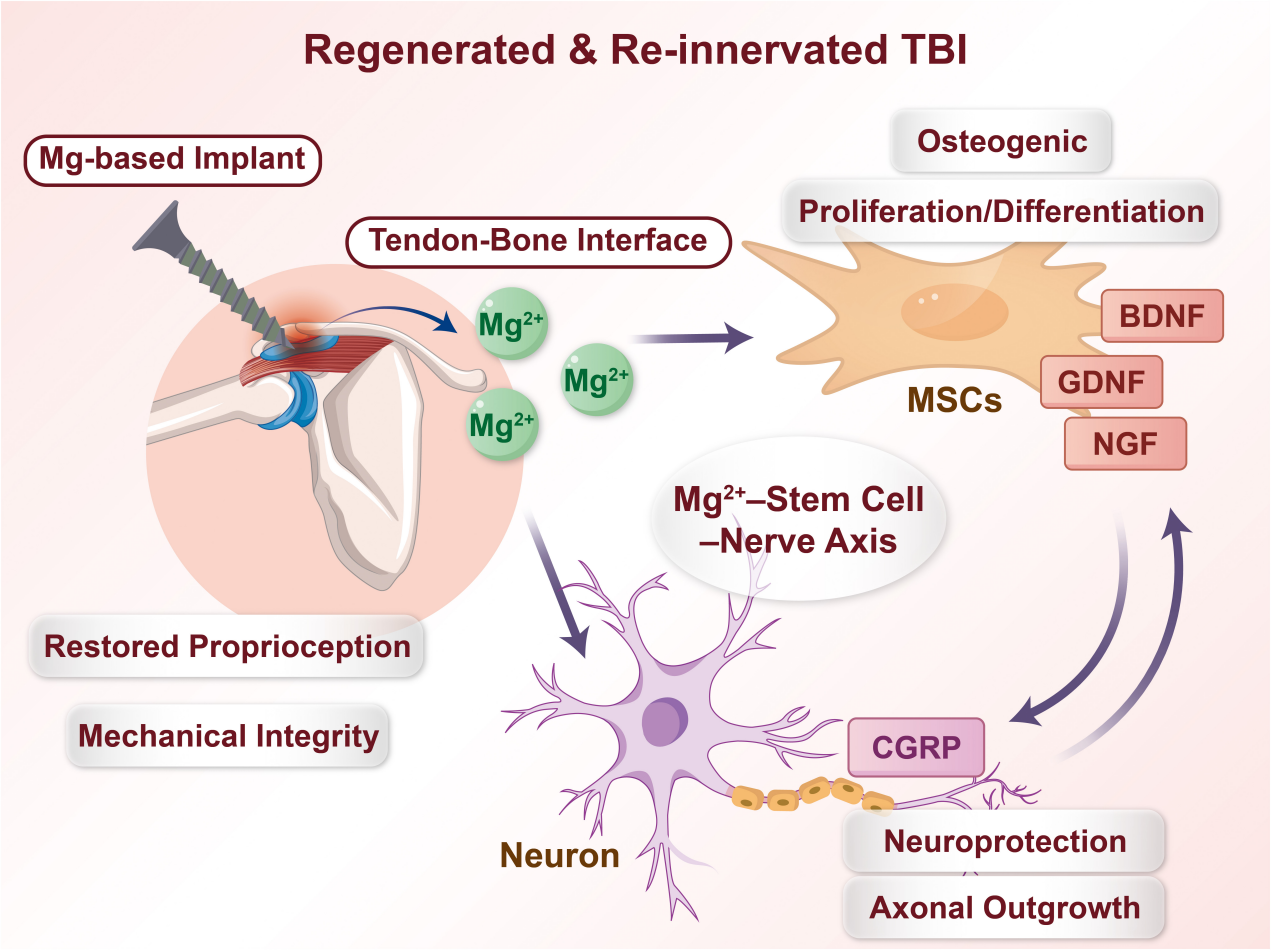
Proposed integrated mechanism of the “Mg^2 +^-Stem Cell-Nerve” axis in neurotized regeneration of the tendon-bone interface (conceptual model).

**FIGURE 3:**
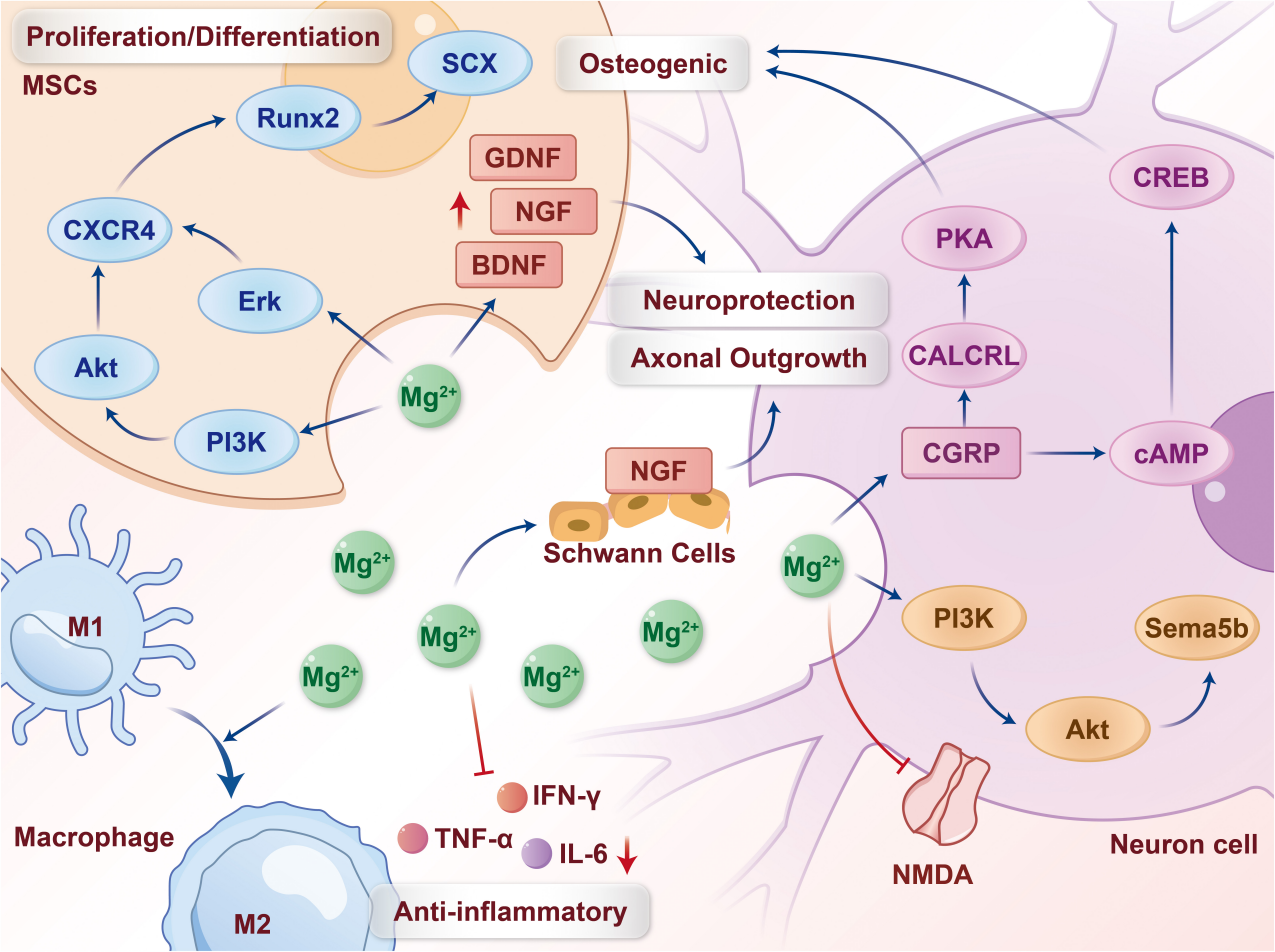
Detailed key cellular and molecular mechanisms underpinning the “Mg^2 +^-Stem Cell-Nerve” axis.

Building upon the multifaceted biological effects of magnesium ions (Mg^2 +^) in regulating stem cell behavior and promoting neural regeneration discussed earlier, this chapter proposes and systematically elaborates the core paradigm driving neurotized repair of the tendon-bone interface –the “Mg^2 +^-Stem Cell-Nerve” Interactive Axis. It should be emphasized that the “Mg^2 +^-Stem Cell-Nerve” axis presented here is a conceptual synthesis derived from integrating evidence across multiple independent studies. While strong experimental support exists for individual interactions (e.g., Mg^2 +^→ MSC, Mg^2 +^→ neuron), direct *in vivo* demonstration of a fully coupled, sequential axis within the same TBI microenvironment is still limited. Thus, the axis should be regarded as a plausible and heuristically valuable model that guides future mechanistic investigation and material design. This axis is not a simple superposition of isolated pathways but an integrative mechanism initiated by the Mg^2 +^ signal, comprising three interlinked and temporally logical axes, which could ultimately form a functional closed loop (see the conceptual model in Figure 2 and the detailed mechanisms in Figure 3 and Table 2). Specifically: Axis I, Mg^2 +^ actively recruits and “empowers” mesenchymal stem cells, laying the foundation for their mediated structural repair and creation of a neurotrophic microenvironment. Axis II, Mg^2 +^ directly activates neurons and Schwann cells, initiating the nerve regeneration program. Axis III, newly formed nerves provide feedback signals that reciprocally regulate stem cell fate and tissue maturation, forming the “Nerve-Stem Cell Unit.” This chapter will elaborate on these three axes following this logical sequence.

**TABLE 2:** Components and functions of the “Mg^2 +^-Stem Cell-Nerve” interactive axis.

Axis component	Key players	Primary functions in TBI regeneration
Signal initiator	Degrading Mg alloy, released Mg^2 +^ ions	Serves as the central bioactive signal; recruits MSC and directly promotes neural regeneration.
Stem cell effector	Mesenchymal stem cells (MSC)	Mediates structural repair (bone/tendon formation); secretes neurotrophic factors (BDNF, NGF, etc.) to support nerve growth.
Neural regenerator	Neurons, axons, Schwann cells (SCs)	Re-establishes neural circuits and proprioception; SCs guide axonal growth and enable re-myelination.
Feedback regulator	Newly formed nerve endings (releasing e.g., CGRP)	Fine-tunes stem cell fate (e.g., promotes osteogenesis via cAMP/CREB); enhances vascularization; drives functional maturation.
Integrated functional unit	Nerve-Stem Cell Unit (axons, SCs, MSC in proximity)	Represents the functional endpoint where structural regeneration and neural innervation are spatially and functionally coupled.

This schematic illustrates the theoretical framework by which Mg^2 +^ released from biodegradable magnesium-based implants may orchestrate synergistic structural and neural repair. The depicted interactions are based on synthesis of evidence from separate studies; their sequential coupling *in vivo* awaits further validation. (A) A magnesium alloy implant at the repair site continuously degrades and releases Mg^2 +^ (green spheres). (B) Mg^2 +^ exerts its effects through three parallel and interconnected axes: (Axis I) Recruiting and activating mesenchymal stem cells (MSC), prompting them to secrete various neurotrophic factors (e.g., BDNF, NGF), creating a microenvironment conducive to nerve regeneration; (Axis II) Directly promoting neuronal axonal growth and the proliferation and functional activation of Schwann cells (SCs), driving nerve fiber regeneration; (Axis III) Modulating the local immune response, promoting macrophage polarization toward the anti-inflammatory M2 phenotype. (C) The above processes synergistically contribute to the formation of the “Nerve-Stem Cell Unit”: Newly formed nerve endings release neuropeptides (e.g., CGRP), which in turn feedback to further regulate MSC osteogenic differentiation and angiogenesis, thereby establishing a self-reinforcing cycle, could potentially achieve dual functional healing of the tendon-bone interface at both structural and reinnervation levels.

This figure details the potential cellular and molecular events involved in the conceptual framework of Figure 2. (Left) Mechanisms related to Axis I (Stem Cell Axis): Mg^2 +^ activates pathways such as PI3K/Akt and Erk, upregulates CXCR4 expression to promote MSC migration, and modulates key transcription factors like Runx2 (osteogenic) and SCX (tenogenic) to guide their differentiation; activated MSC secrete neurotrophic factors such as BDNF, NGF, and GDNF. (Middle) Mechanisms related to Axis II (Neural Axis) and immunomodulation: Mg^2 +^ directly promotes neuronal axonal growth and activates Schwann cells to support re-myelination. Concurrently, Mg^2 +^ exerts neuroprotection by antagonizing NMDA receptors and shapes an anti-inflammatory microenvironment favorable for neural survival and regeneration by modulating macrophage polarization (promoting M1-to-M2 transition) and downregulating pro-inflammatory factors such as TNF-α, IL-6, and IFN-γ. Mechanisms related to Axis III (Feedback Axis): CGRP released from new nerves feedback-promotes osteogenic differentiation and angiogenesis by activating pathways including CALCRL/PKA and cAMP/CREB in MSC and vascular endothelial cells. This figure integrates evidence from different research systems; the spatiotemporal coordination and dominant sequence of these pathways within the complex *in vivo* environment are key foci for future mechanistic research, and their operation as a complete closed loop requires further validation.

### 4.1 Axis I: Mg recruits and “empowers” stem cells, paving the way for nerve regeneration

Mesenchymal stem cells possess the ability to migrate toward injury sites, a biological behavior termed the homing mechanism (66). This process is mediated by various inflammatory or chemotactic factors (67). For example, Vascular Endothelial Growth Factor (VEGF) and Hepatocyte Growth Factor (HGF) released at the injury site can attract MSC (68). Furthermore, the SDF-1α/CXCR4 axis plays a crucial role in MSC homing (69, 70). Other factors positively influencing MSC migration include Substance P and Granulocyte Colony-Stimulating Factor (G-CSF) (71, 72). Studies show that activating intracellular Akt and Erk signaling pathways significantly increases the expression of membrane proteins involved in cell migration (e.g., Aquaporin 1 and CXCR4) on MSC, thereby enhancing their migratory capacity (73).

Magnesium ions generated from the degradation of magnesium implants leads to a significant increase in the expression of TGF-β1 and Platelet-Derived Growth Factor-BB (PDGF-BB) in surrounding tissues, providing direct chemotactic signals for MSC migration toward the injured area (73). These mechanisms collectively promote the migration of MSC to the tendon-bone interface lesion, supplying “seed cells” for repair.

Mesenchymal stem cells that have migrated to the injury site, once “empowered” by Mg^2 +^, efficiently secrete a variety of neurotrophic factors such as BDNF, GDNF, NGF, NT-3, CNTF, and bFGF (74–78). These factors not only prevent neurodegeneration and support neurogenesis and re-myelination (79–84) but also create a local microenvironment rich in neurotrophic factors. Ultimately, concentration gradients formed by these neurotrophic factors can guide the directional growth of nerve axons toward the injured area, establishing a complete regulatory cascade: “Mg^2 +^- MSC – Neurotrophic Microenvironment – Directed Axonal Regeneration.” This provides dual support for neural regeneration and functional repair of the tendon-bone interface (85, 86).

### 4.2 Axis II: Mg directly activates the neural regeneration program

As described in Section “3 Direct and indirect roles of magnesium ions in nerve regeneration,” Mg^2 +^ acts directly on neurons and Schwann cells to initiate the regeneration program. Furthermore, Mg^2 +^ can also influence neural stem/progenitor cells, promoting their differentiation toward neurons via pathways like ERK/CREB, ensuring the operation of the neural regeneration cascade from multiple aspects (42).

### 4.3 Axis III: neo-innervation feedback regulates stem cell fate and tissue maturation

During tendon-bone interface regeneration, newly formed nerve endings are not merely passive endpoints of repair but active participants in regulating tissue maturation through the release of specific neural signals. Key neuropeptides, such as Calcitonin Gene-Related Peptide (CGRP), can bind to their corresponding receptors expressed on mesenchymal stem cells, precisely modulating their lineage-specific differentiation fate.

Calcitonin Gene-Related Peptide is a short neuropeptide with two main forms (α and β), associated with C and Aδ sensory fibers, and distributed primarily in the central and peripheral nervous systems (87). Zhang et al. proposed that the beneficial osteogenic effects of Mg-based implants are largely mediated by CGRP (86). CGRP is a neuropeptide that promotes osteogenic differentiation of precursor cells by activating the cAMP/CREB pathway. In the presence of elevated Mg^2 +^ concentrations, CGRP release from the periosteum is significantly enhanced (88, 89). CGRP has been shown to promote osteogenic differentiation of B MSC and PDSCs, inhibit osteoclastogenesis, and stimulate mesenchymal stem cell (MSC) migration to fracture sites (88, 90).

It should be noted that the majority of evidence for the CGRP-mediated feedback loop derives from bone fracture healing models, particularly the work of Zhang et al. demonstrating that Mg^2 +^-induced CGRP release from periosteal sensory nerves promotes osteogenic differentiation (88, 89). Direct evidence for this mechanism within the TBI microenvironment remains limited, although recent work by Zhao et al. suggests that CGRP receptor activation can enhance tendon-bone healing, providing preliminary support for the relevance of this pathway (4).

In recent years, growing evidence indicates that signaling initiated by α-CGRP significantly influences bone regeneration (91). Studies have also reported that Mg^2 +^ released from implants enters sensory nerve fiber endings, stimulating dorsal root ganglia to release α-CGRP, which subsequently promotes the osteogenic function of MSC via the cAMP/CREB signaling pathway (88). Furthermore, recent research finds that CGRP can also regulate MSC osteogenic differentiation and H-type vessel angiogenesis through the CALCRL/PKA pathway, forming a “neuro-vascular-bone” collaborative regeneration network. Research by Wang Jiali and colleagues further confirmed that activating CGRP receptor signaling via Calcrl overexpression promotes the secretion of factors like SHH and SLIT3, significantly enhancing nerve density and biomechanical properties at the tendon-bone interface (4).

This feedback mechanism prompts the proposal of the “Nerve-Stem Cell Unit” concept, where nerve axons, Schwann cells, and stem cells are in close spatial proximity, forming a functionally integrated dynamic microsystem through paracrine signaling. This unit serves as the biological foundation for achieving structural integration and sensory functional recovery. The establishment of such a functional “Nerve-Stem Cell Unit” represents a plausible outcome based on current evidence, but its spatiotemporal dynamics and causal interdependencies in the healing TBI remain to be fully elucidated. It is worth emphasizing that Mg^2 +^ released from degrading magnesium alloys plays a key integrative role throughout this process: it not only efficiently recruits and activates mesenchymal stem cells initially, creating a neurotrophic factor-rich microenvironment for neural regeneration, but ultimately facilitates the successful establishment of this functional “Nerve-Stem Cell Unit.” This effectively couples structural regeneration with neural innervation, driving regenerated tissue from basic defect filling toward advanced functional maturity.

### 4.4 Complexity and stage-dependence of nerve-immune-bone interactions

The nerve-immune-bone interplay during TBI healing is unlikely to be a simple linear cascade, but rather a dynamic, feedback-rich network with stage-specific characteristics. For instance, while CGRP generally promotes osteogenesis and angiogenesis, its immunomodulatory effects may vary across healing phases–potentially being beneficial during the early inflammatory phase but requiring strict spatiotemporal regulation later. However, whether its overexpression in later stages adversely affects vascularization or impairs fibrotic maturation has not yet been clearly documented (15). Similarly, although M2 macrophage polarization is broadly anti-inflammatory and pro-regenerative, its sustained or excessive amplification may hinder necessary matrix remodeling or mechanical strengthening by suppressing certain fibrogenic pathways (92). Furthermore, it remains unclear whether excessive neurite ingrowth or neuropeptide release could disrupt the orderly collagen alignment essential for biomechanical function. Therefore, the “Mg^2 +^-Stem Cell-Nerve” axis should not be regarded as a fixed pathway but as a tunable system whose output must be precisely matched to the evolving demands of the inflammatory, proliferative, and maturation phases. Future studies should investigate how to adjust Mg^2 +^ release kinetics to optimally navigate these stage-dependent trade-offs.

## 5 Discussion

### 5.1 Comprehensive review of existing evidence and directions for deepening mechanistic research

This review proposes that biodegradable magnesium alloys, through the construction of the “Mg^2 +^-Stem Cell-Nerve” Interactive Axis, offer a potentially innovative theoretical paradigm for advancing tendon-bone interface (TBI) repair from passive structural integration to active neurotized functional regeneration. The core advantage of this paradigm lies in utilizing Mg^2 +^, an endogenous biological signal, to simultaneously initiate two critical pathways: “stem cell-mediated structural reconstruction” and “directed neural regeneration,” achieving functional coupling through the newly formed “Nerve-Stem Cell Unit.” It is essential to clarify that the core bioactive component focused on in this review is magnesium ions (Mg^2 +^), studied primarily in the context of their release from biodegradable magnesium-based metal implants (pure magnesium and magnesium alloys). The vast majority of current basic and preclinical studies use such materials as the source carrier for Mg^2 +^. This framework not only integrates previously isolated cellular and molecular events but also provides clear functional guidance for designing next-generation intelligent biomaterials. However, any emerging theory must undergo rigorous scrutiny of evidence, horizontal comparison, and assessment of translational feasibility on its path to clinical practice (Table 3).

**TABLE 3:** Summary of evidence sources supporting key components of the proposed “Mg^2 +^-Stem Cell-Nerve” axis.

Key component/claim	Evidence source	Representative references
Mg^2 +^ promotes fibrocartilaginous enthesis regeneration in TBI	Direct *in vivo* (rabbit ACL reconstruction)	(18)
Mg^2 +^ promotes MSC osteogenic/tenogenic differentiation	*In vitro*; bone defect models	(37, 38, 73)
Mg^2 +^ enhances MSC migration to injury site	*In vitro*; bone defect models	(38, 73)
Mg^2 +^ promotes axonal outgrowth	Sciatic nerve injury models; *in vitro*	(43, 44)
Mg^2 +^ promotes Schwann cell proliferation and NGF secretion	*In vitro*; sciatic nerve models	(44, 51)
Mg^2 +^ exerts anti-inflammatory effects	*In vitro*; general inflammation studies	(61, 62)
Mg^2 +^ stimulates CGRP release from sensory neurons	Fracture healing model (periosteum)	(88, 89)
CGRP promotes MSC osteogenesis and angiogenesis	*In vitro*; fracture models	(4, 88, 90)
Secreted neurotrophic factors from MSC support nerve regeneration	*In vitro*; spinal cord injury models	(85, 86)

Although numerous *in vitro* and animal studies provide strong support for individual components of the “Mg^2 +^-Stem Cell-Nerve” Axis, we must objectively examine the completeness of its evidence chain. Currently, the most direct evidence centers on the “starting point” (direct regulation of stem cells and neural cells by Mg^2 +^) and the “endpoint” (functional improvement) of the axis. However, there remains a lack of real-time, *in vivo* verification of the dynamic transmission and amplification of signals within the axis – for instance, how the secretome of Mg^2 +^-activated MSC acts on different stages of neural regeneration in a spatiotemporally specific manner within the complex TBI microenvironment. Specifically, several key questions demand further exploration.

It should be emphasized that, although each individual component of the proposed “Mg^2 +^-Stem Cell-Nerve” axis is supported by independent experimental evidence (e.g., Mg^2 +^ promotes MSC osteogenic differentiation and secretion of neurotrophic factors; Mg^2 +^ directly enhances axonal outgrowth and Schwann cell function; CGRP released from nerve endings feedback-regulates bone formation), no single study to date has directly and comprehensively validated the complete sequential operation of this axis as a closed loop within the tendon-bone interface microenvironment. This critical knowledge gap means that the framework proposed herein should be regarded as a testable working hypothesis rather than an established pathway. While the individual components of the proposed axis are supported by robust data from peripheral nerve injury models (for neural effects), bone fracture models (for CGRP-mediated signaling), and *in vitro* stem cell studies (for Mg^2 +^-directed differentiation), their integration into a coordinated, temporally regulated axis within the complex mechanical and cellular environment of the healing enthesis remains to be demonstrated. Future studies employing TBI-specific animal models (e.g., rotator cuff repair or ACL reconstruction in rats, rabbits, or large animals) with targeted interventions–such as selective Mg^2 +^ chelation, pathway inhibition, or cell-type-specific knockout–are needed to validate the causal and sequential relationships proposed here.

Secondly, the dynamic complexity of magnesium alloy degradation cannot be overlooked. Although magnesium alloy degradation is a complex process accompanied by hydrogen gas evolution, local pH changes, and potentially other alloying element ions, existing evidence strongly suggests that the continuously released Mg^2 +^ is the most critical signaling molecule mediating its promotion of tendon-bone interface regeneration, activating multiple pathways such as MAPK/ERK and PI3K/AKT to directly regulate cell fate and function. This also forms the core logic of current research on “Mg^2 +^-stem cell” interactions. Local pH fluctuations and hydrogen gas release accompanying degradation have been shown to negatively impact nerve function. Studies indicate that low pH interferes with sodium/potassium channel function, inhibiting axonal electrical signal conduction and disrupting mitochondrial energy metabolism, thereby exacerbating functional damage in hypoxic nerves hindering the initiation of regeneration (15, 17). Although Mg^2 +^ is regarded as the primary bioactive signal, concomitant local pH fluctuations and hydrogen release may exert counteractive effects on neural cells. For instance, alkaline microenvironments can interfere with axonal electrophysiology and mitochondrial function. Thus, the reported pro-regenerative “net effects” in alloy implantation studies may stem from a balance between beneficial ionic signaling and potentially detrimental degradation chemistry. Future studies should employ models that allow dissociation of ionic versus physicochemical contributions.

Finally, reports on the effects of Mg^2 +^ on specific cells like Schwann cells show inconsistencies, which likely stem from differences in ion release kinetics, culture systems, or animal models used across studies (62). This highlights the importance of promoting standardization and refinement of relevant research models for establishing solid consensus.

### 5.2 Key challenges and solutions for clinical translation

The translational pathway for Mg-based TBI implants faces significant challenges, foremost among which is achieving controllable degradation behavior in the human TBI microenvironment. While progress has been made in bone fixation (e.g., WE43 alloy screws) (93), a systematic review of preclinical studies further supports the potential of magnesium-based fixation for enhancing tendon-graft healing (20). The healing context of the TBI–with its distinct mechanical, vascular, and cellular milieu–therefore requires material solutions specifically designed for the TBI. These challenges can be categorized into three interrelated aspects: degradation behavior, mechanical design, and preclinical validation.

First, degradation behavior must be precisely controlled. Magnesium alloys have a density of 1.74–2.0g/cm^3^ and elastic modulus of 41–45 GPa, close to human bone, and their degradation rate can be tuned through alloying and processing (21). However, rapid degradation can lead to localized hydrogen gas accumulation and local pH elevation, both of which may adversely affect axonal function and delay neural regeneration (17). Optimal degradation profiles for TBI repair remain undefined, but enthesis remodeling typically takes months rather than weeks (1, 5, 6). Alloying elements such as zinc, calcium, or manganese can be incorporated to modulate corrosion rates, but their neurobiological effects must be carefully evaluated. Additionally, the byproducts generated during magnesium degradation warrant attention. Hydrogen evolution and pH elevation are intrinsic to magnesium degradation (21). These byproducts are more challenging in the TBI environment than in bone, as the tendon-bone interface lacks the rich vascular network that rapidly clears degradation products from bone tissue; hydrogen accumulation may form gas cavities, and local pH elevation may affect cell viability and neural electrophysiology (17).

Second, mechanical design must accommodate the unique loading environment of the TBI. The biomechanical demands of TBI healing differ markedly from those of bone fixation. Tendon-bone interfaces are subject to complex tensile, shear, and rotational forces that vary across the healing timeline (2, 94). Implant designs must provide sufficient initial mechanical stability while gradually transferring load to regenerating tissue as degradation proceeds, and a balance between these two aspects is required. Finite element modeling combined with *in vivo* validation in large-animal models is essential to optimize fixation geometry and degradation characteristics.

Third, to address the above challenges, next-generation implants require intelligent design. A shift from “generic” to “precise and intelligent” design is required. Next-generation implants should encompass at least three dimensions: (1) spatiotemporal control – smart materials for sequential release of Mg^2 +^ and auxiliary factors to match different healing phases; (2) physical guidance – macro-to-nano topological structures to guide ordered tendon collagen regeneration and directional axonal extension; (3) biological information – genetic engineering or exosome loading to encode complex signals (85, 86). Integration of these dimensions aims to achieve controllable degradation, spatiotemporal oscillatory release, and topographical guidance.

Collectively, these requirements distinguish TBI repair from conventional bone fixation. Compared with conventional bone fixation, TBI repair faces unique challenges: (i) more complex mechanical environment (multi-axial loading) (2, 94); (ii) longer healing timeline (months vs. weeks) (1, 5); (iii) higher demand for neural regeneration (functional reinnervation) (4, 14); and (iv) lower tolerance for degradation byproducts (neural sensitivity to H_2_ and pH shifts) (17).

Finally, appropriate animal models are essential to validate these designs. While rodent models are suitable for initial mechanistic exploration, large animal models (goat, pig rotator cuff tear, ACL reconstruction) are indispensable for evaluating functional neural innervation and long-term mechanical performance, owing to their closer resemblance to humans in anatomy, load, and healing patterns (94–96). Comprehensive evaluations should include histology, biomechanics, electrophysiology, and behavioral assessments.

## 6 Conclusion

In summary, the magnesium alloy-based TBI repair strategy predicated on the “Mg^2 +^-Stem Cell-Nerve” axis represents an emerging concept that, pending further experimental validation, holds promise for integrating the biological effects of materials with the demands of neurotized regeneration. It transcends the traditional material design philosophy centered on “mechanics,” pioneering a new path of “biological signal-driven functional reconstruction.” Currently, this field is transitioning from proof-of-principle studies toward translational development, but considerable research is still needed to bridge the gap between promising preclinical outcomes and clinical application. Future work should focus on the deep integration of three core directions. First, at the “cognitive” level, employing cutting-edge technologies like single-cell sequencing, *in vivo* imaging, and organoids to analyze the signaling logic of Mg^2 +^ within the complex cellular network of the TBI at finer spatiotemporal scales, and to reveal its dialog with endogenous electrical and mechanical signals. Second, at the “creation” level, promoting interdisciplinary innovation across materials science, engineering, and biology to develop “fourth-generation” intelligent magnesium-based composite materials with sensing, responding, and regulating capabilities, enabling dynamic intervention in the regeneration process. Third, at the “validation” level, establishing standardized preclinical research paradigms and efficacy evaluation systems to lay a solid evidence base for eventual human clinical trials.

Although numerous scientific and engineering challenges lie ahead, through sustained multidisciplinary collaboration, the neurotized TBI repair strategy leveraging magnesium-based materials holds promise to tangibly advance the paradigm shift in the treatment of musculoskeletal injuries – from restoring anatomical structure to reconstructing sensorimotor function – ultimately delivering rehabilitation outcomes with enhanced quality of life for patients.
